# Parental Binge Alcohol Abuse Alters F1 Generation Hypothalamic Gene Expression in the Absence of Direct Fetal Alcohol Exposure

**DOI:** 10.1371/journal.pone.0089320

**Published:** 2014-02-20

**Authors:** Magdalena M. Przybycien-Szymanska, Yathindar S. Rao, Sarah A. Prins, Toni R. Pak

**Affiliations:** Loyola University Chicago Health Science Division, Department of Cell and Molecular Physiology, Maywood, Illinois, United States of America; Sapienza University of Rome, Italy

## Abstract

Adolescent binge alcohol exposure has long-lasting effects on the expression of hypothalamic genes that regulate the stress response, even in the absence of subsequent adult alcohol exposure. This suggests that alcohol can induce permanent gene expression changes, potentially through epigenetic modifications to specific genes. Epigenetic modifications can be transmitted to future generations therefore, and in these studies we investigated the effects of adolescent binge alcohol exposure on hypothalamic gene expression patterns in the F1 generation offspring. It has been well documented that maternal alcohol exposure during fetal development can have devastating neurological consequences. However, less is known about the consequences of maternal and/or paternal alcohol exposure outside of the gestational time frame. Here, we exposed adolescent male and female rats to a repeated binge EtOH exposure paradigm and then mated them in adulthood. Hypothalamic samples were taken from the offspring of these animals at postnatal day (PND) 7 and subjected to a genome-wide microarray analysis followed by qRT-PCR for selected genes. Importantly, the parents were not intoxicated at the time of mating and were not exposed to EtOH at any time during gestation therefore the offspring were never directly exposed to EtOH. Our results showed that the offspring of alcohol-exposed parents had significant differences compared to offspring from alcohol-naïve parents. Specifically, major differences were observed in the expression of genes that mediate neurogenesis and synaptic plasticity during neurodevelopment, genes important for directing chromatin remodeling, posttranslational modifications or transcription regulation, as well as genes involved in regulation of obesity and reproductive function. These data demonstrate that repeated binge alcohol exposure during pubertal development can potentially have detrimental effects on future offspring even in the absence of direct fetal alcohol exposure.

## Introduction

Several decades of research have established that maternal alcohol consumption during critical periods of fetal brain development leads to devastating long-term consequences on cognitive function and social behavior. However, less is known about the consequences of parental alcohol consumption, outside of gestational periods, on alcohol-dependent epigenetic modifications that could subsequently manifest as detrimental phenotypic changes in the offspring. Importantly, transgenerational effects, presumably transmitted through the germline, have been reported after exposure to a variety of physiological insults including endocrine disruptors [1], [2], stress [3], [4], [5] and fetal alcohol exposure [6]. In this study, we examined gene expression profiles in the hypothalamus of offspring (F1, never alcohol exposed) whose parents were exposed to alcohol during a restricted period of time (adolescence) and then compared them to animals whose parents were never exposed to alcohol.

Alcohol is the most commonly abused drug by youth under 21 years of age and, according to the Center for Disease Control and Prevention (CDC), 11% of all alcohol in the United States is consumed by youth between the ages of 12 and 21. More importantly, greater than 90% of alcohol consumed by youth is achieved through “binge” drinking, which is defined by the National Institute on Alcohol Abuse and Alcoholism (NIAAA) as consuming enough alcohol in two hour period to raise the blood alcohol concentration (BAC) above the 0.08% legal driving limit. Binge drinking during adolescence has been linked with a greater risk for alcoholism, drug abuse, depression, and suicide [7].

Extensive neurological changes are manifested during adolescence including increased neurogenesis and neuronal spine density, synaptic pruning, and altered neuronal activity patterns [8], [9], [10], [11], [12], [13], thereby making it a particularly vulnerable developmental time period for the negative effects of alcohol. Previous studies have demonstrated that adolescent brain development is impaired by both physical and psychological stressors, including alcohol [14], [15], [16]. Our lab and others have shown that binge alcohol exposure, specifically during adolescence, had long-lasting effects on the expression of genes that regulate the stress response, even in the absence of subsequent adult alcohol exposure [14], [17], [18]. These results raised the possibility that alcohol exposure during adolescence can induce epigenetic modifications that result in permanent phenotypic changes in adult gene expression patterns and could potentially be transmitted to future generations.

In these studies we examined the gene expression profile in the hypothalamus of F1 generation offspring whose parents were exposed to binge alcohol during adolescence. The hypothalamus is a critical central regulator of multiple physiological processes including the stress response, appetite/satiety, circadian rhythms, osmoregulation, blood pressure, and reproduction. Strikingly, our results showed that the hypothalamus of offspring from alcohol-exposed parents had significant differences in genes that mediate neurogenesis and synaptic plasticity during neurodevelopment, genes important for directing chromatin remodeling, posttranslational modifications or transcription regulation, as well as genes involved in regulation of obesity and reproductive function. These results were highly compelling and, to our knowledge, are the first to demonstrate that adolescent binge alcohol exposure outside of gestational periods has an effect on hypothalamic gene expression in the F1 offspring. Taken together, these data illustrate the important novel concept that adolescent alcohol exposure can have detrimental effects on future offspring even in the absence of direct fetal alcohol exposure.

## Methods

### Ethics Statement

All animal procedures were approved by the Loyola University Medical Center Institutional Animal Care and Use Committee (IACUC), Loyola University Chicago permit #2011002.

### Animals

**Figure 1:**
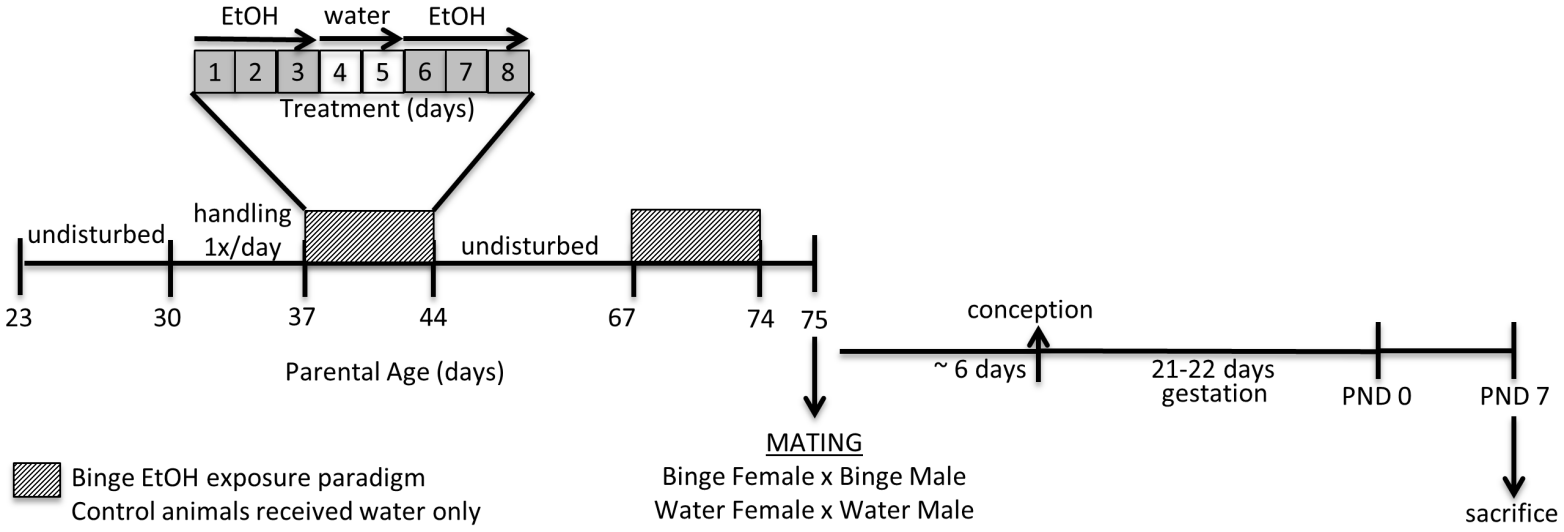
Adolescent binge EtOH exposure paradigm. Diagram depicting the experimental design. Male and female Wistar rats were purchased from Charles River Laboratories at weaning (PND 23). Animals were handled for 5 min/1x/day beginning on PND 30. Hatched box indicates treatment days for the binge EtOH exposure paradigm. Control rats were given tap water once/day during the 8-day treatment period. Rats were mated according to treatment groups on PND 75. Offspring were untreated and sacrificed at PND 7.

Male and female Wistar rats were purchased from Charles River Laboratories (Wilmington, MA) at weaning (postnatal day (PND) 23) and allowed to acclimate for 7 days after arrival. Animals were handled for 5 min./once/day beginning at PND 30. Pubertal EtOH exposure began on PND 37, which is defined as peri-puberty [19], [20], [21]. Animals were undisturbed following the first exposure of our binge EtOH exposure paradigm until PND 68 (late puberty/early adult) at which time they received a second exposure to the same treatment paradigm (Fig. 1). During the duration of the experiment, males and females were separately housed in pairs on a 12∶12 light/dark cycle with lights on at 0700 h with food and water available *ad libitum.*

### Binge Exposure Paradigm and Treatment Design

Rats were handled 5 min./once/day for 7 d prior to treatment to control for nonspecific stress responses. At 37 d, animals were given 3 g/kg EtOH (20% v/v in tap water; N = 3/sex), or tap water alone (N = 3/sex), once/day via oral gavage at 10∶00 AM to avoid disrupting normal feeding patterns. This process was repeated according to the following schedule for a total duration of 8 consecutive days: 3 d EtOH, 2 d tap water, 3 d EtOH. Control animals were given tap water alone once/day for 8 consecutive days (Fig. 1). Our previous studies showed that this repeated binge-pattern EtOH paradigm does not affect body weight/growth curves and consistently results in blood alcohol concentrations (BAC) of 150–180 mg/dl in males and 210–240 mg/dl in females [14], [22], [23]. We and others have previously used this paradigm as a model for the pattern of binge alcohol consumption observed in adolescents [14], [22], [24], [25] and BAC achieved are similar to those observed in humans following a binge drinking episode [26], [27]. After peri-pubertal treatments, animals were left undisturbed in their home cage until PND 68 when each group was again exposed to their respective treatment (i.e. control or binge EtOH, Fig. 1). We waited 24 hours after the last dose of EtOH to ensure that blood alcohol concentrations in the parents were undetectable at the time of mating (data not shown). Animals were grouped into mating pairs: binge male+binge female (N = 3 pairs); water male+water female (N = 3 pairs). All of the females gave birth to 12–16 pups approximately 28 d after being housed with a male, indicating that conception took place approximately 6 d after pairing; therefore, the pups were never directly exposed to alcohol at any time. The pups were culled to 10 pups/litter immediately after birth in an equivalent sex ratio. The pups were then returned to their biological mothers until PND 7 at which time they were deeply anesthetized on ice and sacrificed (Fig. 1). Brains were rapidly removed, the hypothalamus microdissected on ice, and then stored in −80°C until further processing for a genome-wide analysis on hypothalamic total RNA samples using a chip-based microarray (Southern California Genotyping Consortium, SCGC, Illumina Rat Ref-12). The PND 7 time point was chosen because the extent of rat neurodevelopment at PND 7 is roughly equivalent to that of a human infant at birth [28].

### Sample Processing, Microarray Analysis and Bioinformatics

Total RNA from PND 7 rat pup hypothalami (N = 6/treatment/sex) was isolated using RNeasy Plus Mini Kit (Qiagen, Inc.) according to manufacturer’s instructions and submitted to the Southern California Genotyping Consortium (SCGC) for microarray processing. The samples were loaded onto a RatRef-12 expression BeadChip (Illumina) which contained 22,523 probes targeted to 21,910 unique genes that were selected from the National Center for Biotechnology Information (NCBI) Reference Sequence (RefSeq – Release 16) database. Total RNA purity and integrity were verified by SCGC. Multiple control probes on the chip were used to account for variation in chip production, sample labelling and assay conditions. Raw data were processed by subtraction of background signals and signal intensities above threshold (>500) were analyzed using GenomeStudio V2010.3 software. A differential score was calculated for each gene in order to determine statistical significance between treatment groups (p<0.05 by one-way ANOVA). All of the statistical analyses were performed by the Biostatistics Core Facility at Loyola University Stritch School of Medicine in consultation with Dr. James Sinacore. Of the 21,910 genes targeted on the microarray chip, 11,494 and 11,503 were detected above threshold in the male and female hypothalamus, respectively. Further analysis of these genes was performed using DAVID Bioinformatics Resources 6.7 (National Institute of Allergy and Infectious Diseases (NIAID), NIH) to group the genes into functional categories [29].

### Quantitative Real-time PCR (qRT-PCR)

The same total RNA samples used for the microarray analysis were then subjected to further analysis using qRT-PCR (N = 6/sex/treatment group). Tissue collection and qRT-PCR were performed as previously reported [25]. Briefly, 0.5 µg total RNA was reverse transcribed using the First Strand Synthesis SuperMix for qRT-PCR (Invitrogen Inc., Carlsbad, CA). Roche FastStart SYBR Green Master Mix was added to specific upper and lower primers at 0.25 µM final concentration (see primer sequences in Table 1). Next, 2 µL cDNA templates were added to duplicate reactions performed in 96 well plates. Quantification of the target gene expression was achieved by extrapolating from standard curve of known concentrations of the hypoxanthine guanine phosphoribosyl transferase 1 (HPRT) housekeeping gene ran simultaneously in the same plate. A second housekeeping gene that has higher constitutive expression than HPRT, Glyceraldehyde 3-phosphate dehydrogenase (GAPDH) was also ran on the same plates. The samples were normalized to HPRT and GAPDH separately, as well as to the geometric mean of the combined housekeeping genes. Our results showed that HPRT and GAPDH did not change between treatment groups and the results were the same independent of whether the genes of interest were normalized to the individual housekeeping genes, or the geometric mean of both genes. All samples were quantified using the ΔΔCt method as described previously [30]. Statistical significance between treatment groups (p<0.05 by one-way ANOVA). All of the statistical analyses were performed by the Biostatistics Core Facility at Loyola University Stritch School of Medicine in consultation with Dr. James Sinacore.

**Table 1:** Primer sequences for selected genes.

GENENAME	UPPER PRIMER	LOWER PRIMER
**APOE**	5′GTTGTTTCGGAAGGAGCTGGT	5′CCTGTCAGCAATGGGACCAA
**BMP1**	5′ACAGTGAGAGCAGCAACCTC	5′TGCCAAATGTGTTCACGCAG
**DICER1**	5′GGGAAAGTCTGCAGAACAAAC	5′GGCTGTCTGAGGTCTTAGTTC
**DNMT 1**	5′CCCTGATCCATTTGGCTGGT	5′TCTGCCCGTTCTTGTCTTCC
**EGR2**	5′ACTACATCAGCAACTCCTGGC	5′GCCTTGGCGGTCATCATTTG
**FGF13**	5′TCTTCGGGTGGTGGCTATTC	5′ATCGGGAGAACTCCGTGAG
**FGFR3**	5′GAGACTTGGCTGCCAGAAAC	5′GGAGGACACCAAAAGACCA
**GNRH**	5′CTGCTGACTGTGTGTTTGGAAGG	5′CCTGGCTTCCTCTTCAATCA
**HDAC3**	5′ACACCCGATGAAACCCCATC	5′TCAGAATGGAAGCGGCACAT
**IGF2R**	5′CATGATGGGTCCAAGGCAGT	5′GAAAGGTGGGCAGGCATACT
**PAK3**	5′TCCTCGGGATGGATGGTTCT	5′AGCTGACAGTCTCTCGGGAT
**RELN**	5′ACAAACCTGACAGCCGAGAG	5′CACACACGTTCCTTGTGCAG
**SERPINI1**	5′AGCCGAGTGGTCAGTGAATG	5′ACTTGGTAGATGCCACCAGC
**SIRT2**	5′GCTCGCACTCGCTACCTTAT	5′AGCAGACGTGGTTACAGTGG
**SUMO2**	5′CCTCTTTTGTGAAGCGGCAG	5′TCCTCCATTTCCAACTGTCGT
**VAMP3**	5′CGCCGCCAAAATGTCTACAG	5′TTGTTTCAAACTGCGAGGCG
**WBP4**	5′GAGTGGCCCCAGTCTTCAAA	5′CTTGGAAAGGACACGCCTCA

Intron-spanning primers were designed to detect selected genes that were initially identified by microarray analysis.

## Results

### Global Microarray Analysis

We used a genome-wide microarray approach to determine the effects of parental adolescent binge EtOH exposure on transgenerational (F1, EtOH naïve) gene expression in the hypothalamus. This approach was used as an initial screen to identify specific genes of interest, which were then validated using qRT-PCR on hypothalamic samples from the same animals. In this report, qRT-PCR data rather than raw microarray data are described. The complete microarray dataset was submitted to the Gene Expression Omnibus database (accession #GSE53028). Mating pairs (both male and female partners) were exposed to water alone or to our binge EtOH paradigm. Importantly, the mating pairs (parental generation) had undetectable BAC at the time of mating and the females were not exposed to EtOH during gestation; therefore, the offspring were never directly exposed to EtOH at any time. There were no differences in the time to fertilization (approx. 6 days), total number of pups (12–16), birth weights, or male:female sex ratios in either treatment group (data not shown). Moreover, all pups were viable and did not exhibit any overt abnormalities within the first week of postnatal life. Overall, 16.8% more genes were significantly altered in females (767 genes) compared to males (638 genes), despite only a 0.7% sex difference in the total number of genes detected (11,503 female and 11,494 male). Interestingly, gene expression patterns in the offspring were overall decreased (77.2%) due to parental adolescent binge EtOH exposure, compared with 22.8% of genes that were increased. Specifically, parental adolescent binge EtOH exposure significantly decreased 602 genes in female pups and 482 in males, while only 165 and 156 genes were increased in females and males, respectively.

### Overall Analysis of Functional Gene Clusters

To determine the functional significance of the genes that were detected by microarray analysis as being altered by parental adolescent binge EtOH exposure, we performed a functional gene cluster analysis using the Database for Annotation, Visualization and Integrated Discovery (DAVID) bioinformatics resources 6.7 software [29], [31]. Using this software, we were able to group genes that have related biological functions into distinct clusters. This allowed us to make predictions about the possible functions of the hypothalamus that could be altered due to parental binge EtOH exposure. The gene groups that came up (increased/decreased) with the analysis as a result of parental binge EtOH exposure fell broadly into 19 functional gene cluster categories. Notably, genes that were significantly decreased by parental binge EtOH exposure were restricted to fewer functional clusters (8 clusters) than the genes that were increased (14 clusters, Table 2), despite the fact that the majority of genes significantly altered according to the microarray analysis were decreased (77.2% compared to 22.8% increased). Moreover, distinct functional gene clusters for genes that were decreased were more widely represented in female pups compared to males. For instance, there were no gene clusters unique to males for the downregulated genes, yet 5 were unique to females and 3 were common between both males and females. By contrast in the 14 clusters representing genes that were upregulated, 5 were unique to males, 2 were unique to females, and 7 were common between both sexes. In addition, several multifunctional genes in both males and females could not be grouped into a distinct functional cluster.

**Table 2:** Gene cluster analysis using DAVID software.

GENE CLUSTER	# GENES DECREASED MALE OFFSPRING	# GENES DECREASED FEMALE OFFSPRING
Ribosomal proteins	109	87
Cell cycle	15	80
mRNA processing/splicing	10	11
Transcriptional regulation	–	60
Cell signaling/kinases	–	43
Disulfide bonds	–	26
Glycoproteins	–	33
G-protein coupled receptors	–	11
**GENE CLUSTER**	**# GENES INCREASED MALE OFFSPRING**	**# GENES INCREASED FEMALE OFFSPRING**
Ribosomal proteins	16	14
Cell cycle	–	–
mRNA processing/splicing	–	–
Transcriptional regulation	31	–
Cell signaling/kinases	13	55
Disulfide bonds	7	3
Glycoproteins	10	5
G-protein coupled receptors	–	–
Metal ion binding	28	35
Secreted proteins	4	3
Cell adhesion	10	–
EGF-like domains	–	3
ATP-binding	11	–
Nucleotide binding	13	–

The total number of genes identified by microarray analysis as significantly altered in the offspring due to parental adolescent binge EtOH exposure. Genes are grouped according to unique functional gene clusters.

### Functional Cluster Analysis in Male Offspring

There were a total of 3 functional gene clusters that included all of the genes downregulated in male offspring (Table 2). The most highly represented of these was a cluster of genes that encoded ribosomal proteins (109 genes). The other 2 clusters contained genes encoding molecules involved in cell cycle (15 genes) and mRNA processing/splicing (10 genes). In general, there were more widely diverse gene clusters associated with the genes that were upregulated in males compared to those that were downregulated (Table 2). Highly represented in this group were clusters that included genes encoding nuclear proteins (57 genes), kinases (48 genes) and molecules involved in metal ion binding (35 genes). A minor representation of clusters included genes for ribosomal proteins (14 genes), disulfide bonds (3 genes), glycoproteins (5 genes), signaling molecules (7 genes), secreted proteins (3 genes) and EGF-like domains (3 genes).

### Functional Cluster Analysis in Female Offspring

Overall, more total genes were altered by parental binge EtOH exposure in the F1 generation PND 7 females compared to males (767 genes and 638 genes, respectively). Global gene changes in females could be clustered into 13 distinct functional groups, of which 8 contained genes that were significantly downregulated in females (Table 2). These clusters contained genes encoding ribosomal proteins (87 genes), cell cycle proteins (80 genes), signaling proteins (43 genes), G protein-coupled receptors (11 genes) and proteins involved in transcriptional regulation (60 genes), mRNA processing/splicing (11 genes), disulfide bonds (26 genes) and glycoproteins (33 genes). Genes that were upregulated in females could be grouped into 9 clusters (Table 2) including nuclear proteins (57 genes), metal ion binding (35 genes), kinases (48 genes), ribosomal proteins (14 genes), EGF-like domains (3 genes) secreted proteins (3 genes), signalling molecules (7 genes), disulfide bonds (3 genes) and glycoproteins (5 genes).

### qRT-PCR Validation of Selected Genes from Functional Clusters Identified using DAVID Software

**Figure 2:**
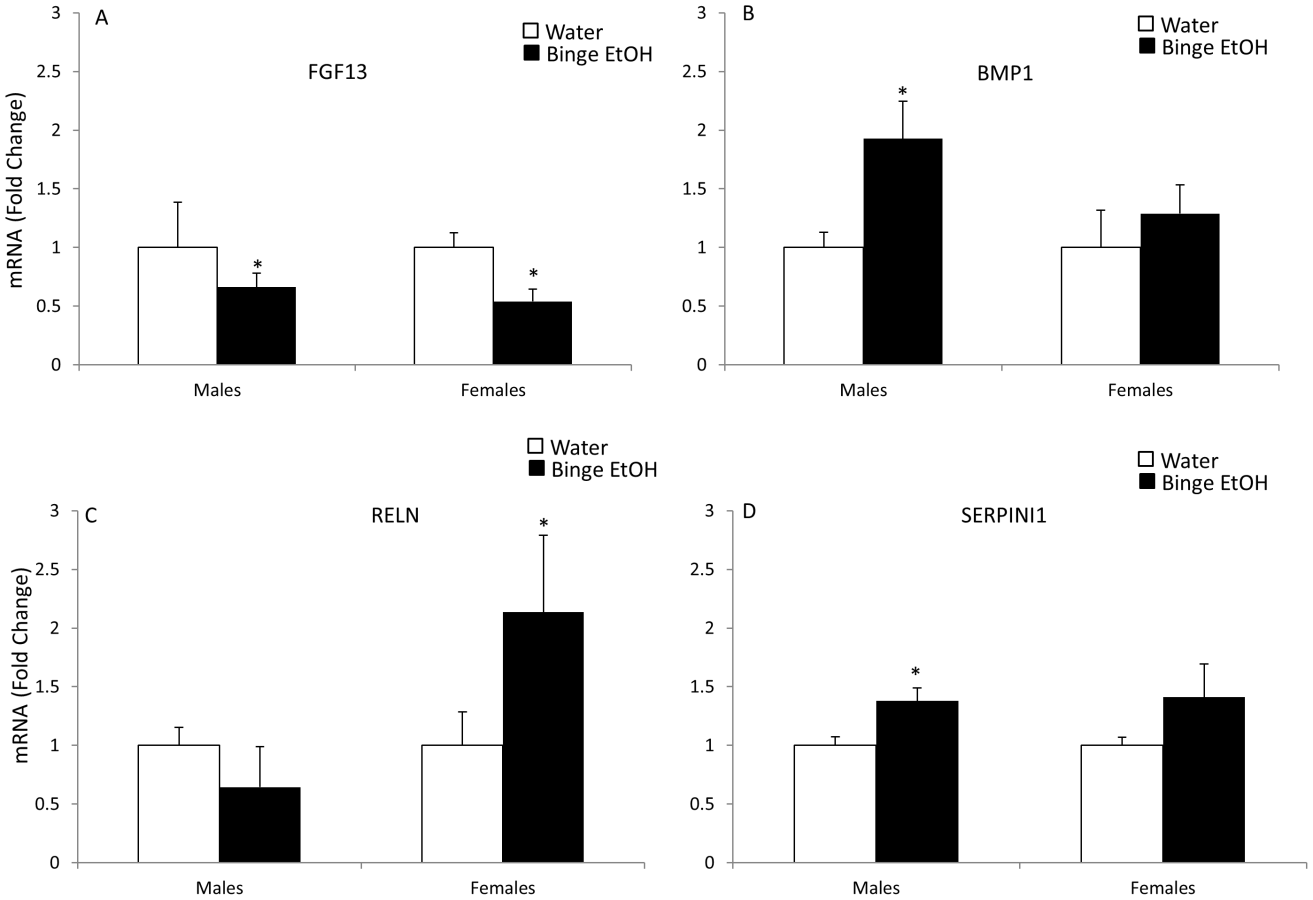
Effects of parental adolescent binge EtOH exposure on the expression of hypothalamic genes involved in neurodevelopment in the hypothalamus of PND 7 male and female offspring. qRT-PCR analysis of FGF13 (A), BMP1 (B), Reln (C) and Serpini1 (D) mRNA expression in the hypothalamus of PND 7 male and female pups from water- or binge EtOH-treated parents (white and black bars, respectively). Data are expressed as mean ± SEM of mRNA fold change in relation to control pups from water-treated parents. (*) indicates a statistically significant difference from control (p<0.05).

**Figure 3:**
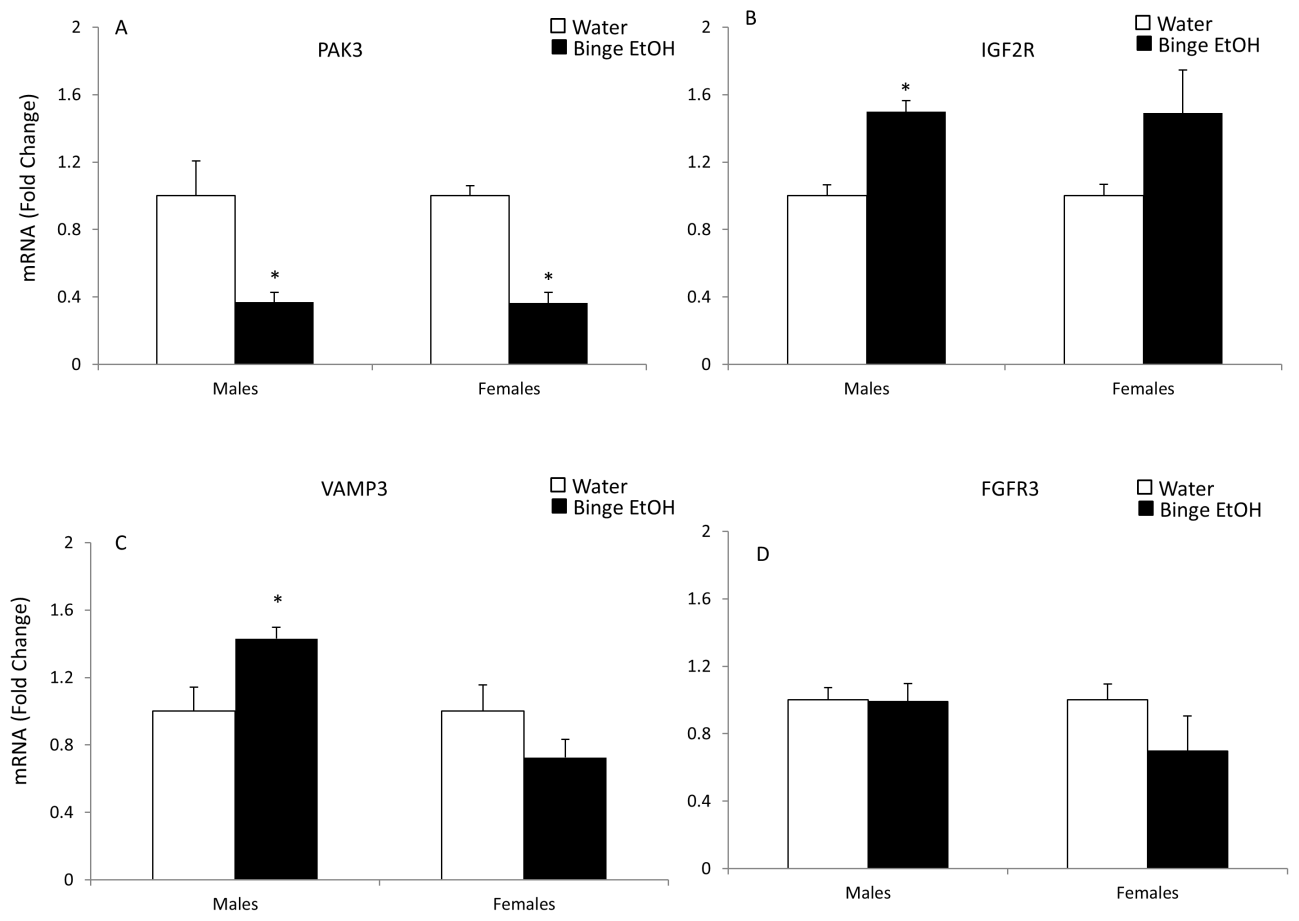
Effects of parental adolescent binge EtOH exposure on the expression of hypothalamic genes involved in synaptic plasticity in the hypothalamus of PND 7 male and female offspring. qRT-PCR analysis of PAK3 (A), IGF2R (B), VAMP3 (C) and FGFR3 (D) mRNA expression in the hypothalamus of PND 7 male and female pups from water- or binge EtOH - treated parents (white and black bars, respectively. Data are expressed as mean ± SEM of mRNA fold change in relation to control pups from water- treated parents. (*) indicates a statistically significant difference from control (p<0.05).

**Figure 4:**
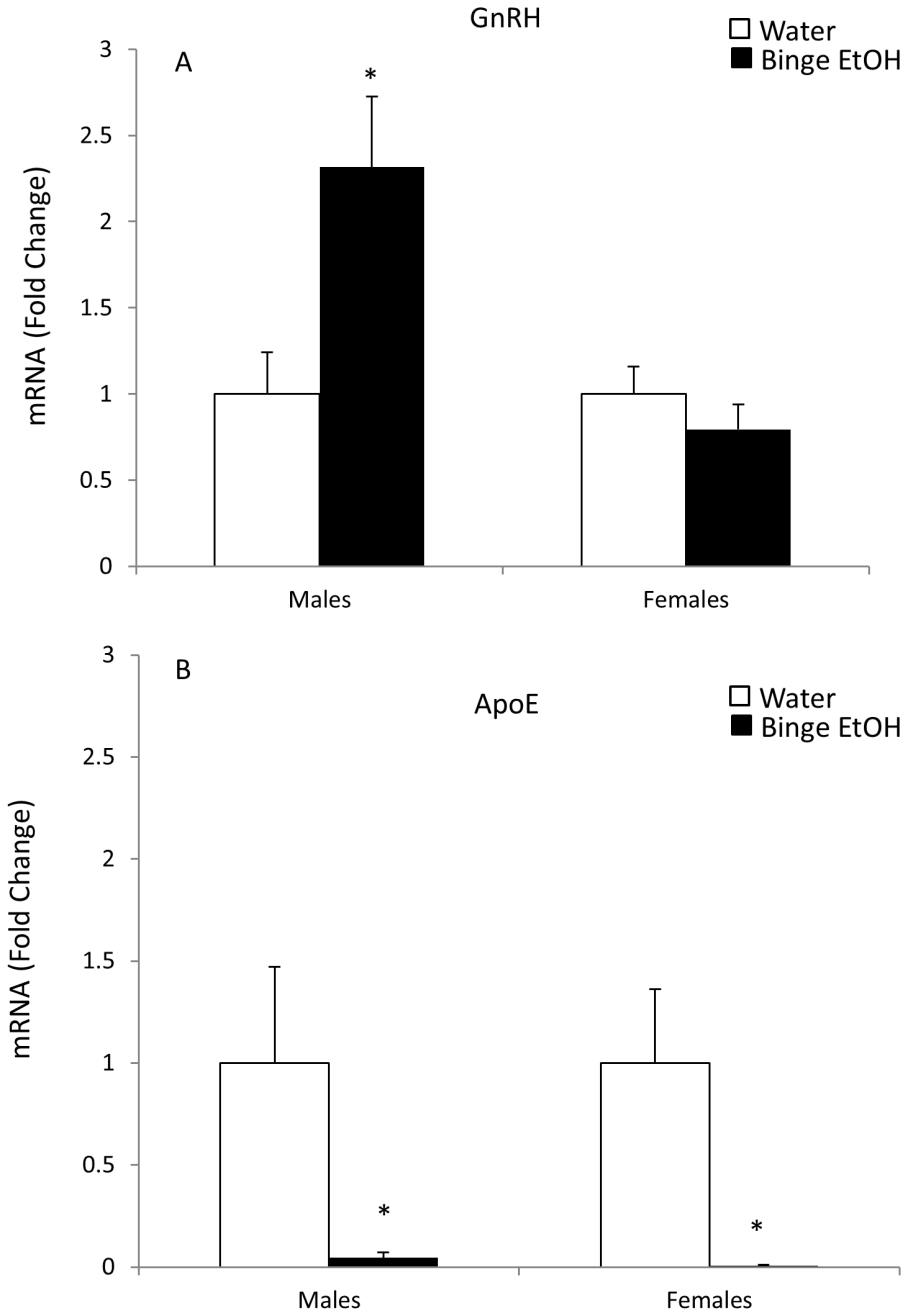
Effects of parental adolescent binge EtOH exposure on the expression of hypothalamic genes involved in metabolic functions in the hypothalamus of PND 7 male and female offspring. qRT-PCR analysis of GnRH (A), ApoE (B) mRNA expression in the hypothalamus of PND 7 male and female pups from water- or binge EtOH - treated parents (white and black bars, respectively). Data are expressed as mean ± SEM of mRNA fold change in relation to control pups from water- treated parents. (*) indicates a statistically significant difference from control (p<0.05).

**Figure 5:**
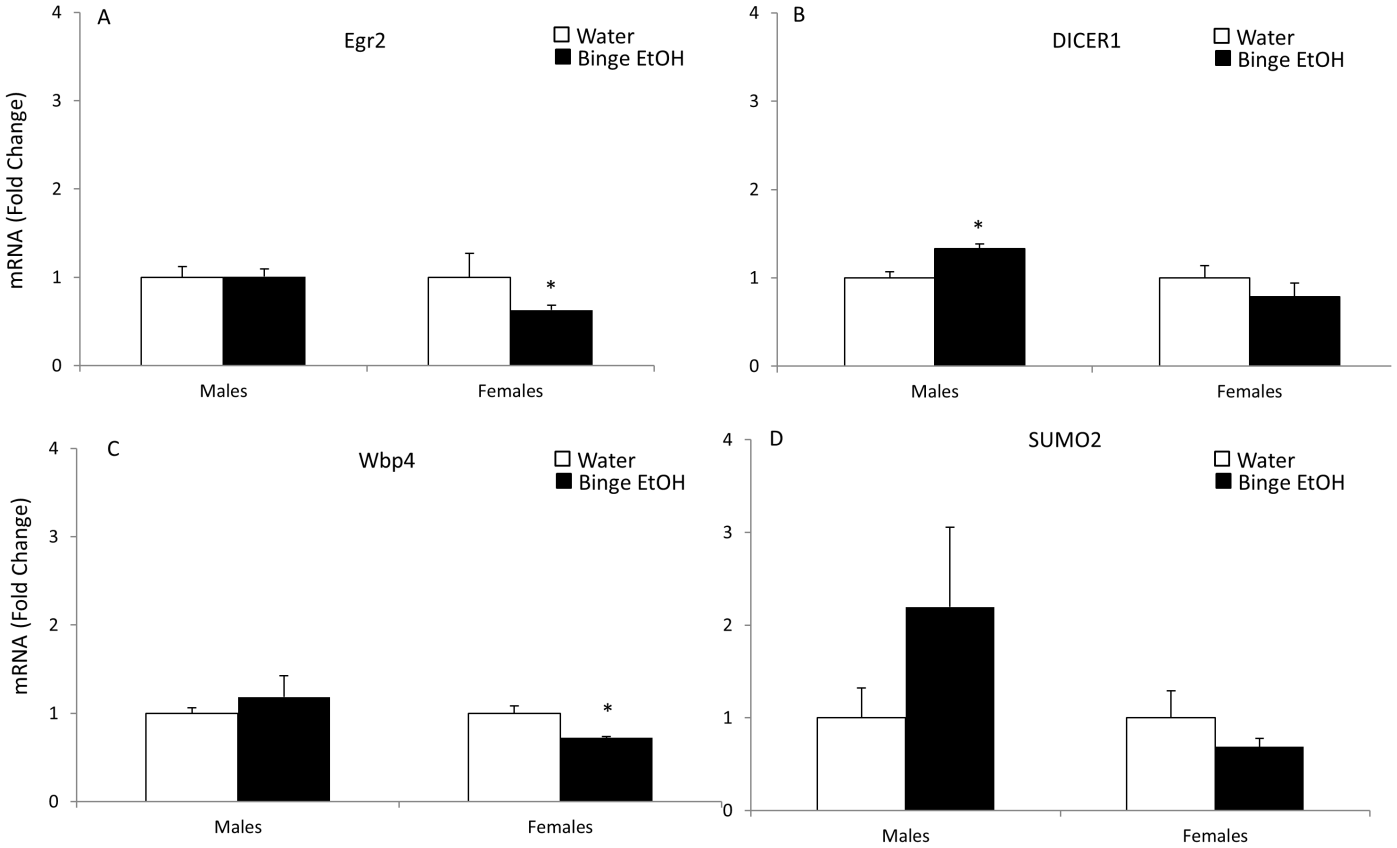
Effects of parental adolescent binge EtOH exposure on the expression of hypothalamic genes involved in transcription and translational regulation in the hypothalamus of PND 7 male and female offspring. qRT-PCR analysis of Egr2 (A), DICER1 (B), Wbp4 (C) and SUMO2 (D) mRNA expression in the hypothalamus of PND 7 male and female pups from water- or binge EtOH - treated parents (white and black bars, respectively). Data are expressed as mean ± SEM of mRNA fold change in relation to control pups from water- treated parents. (*) indicates a statistically significant difference from control (p<0.05).

**Figure 6:**
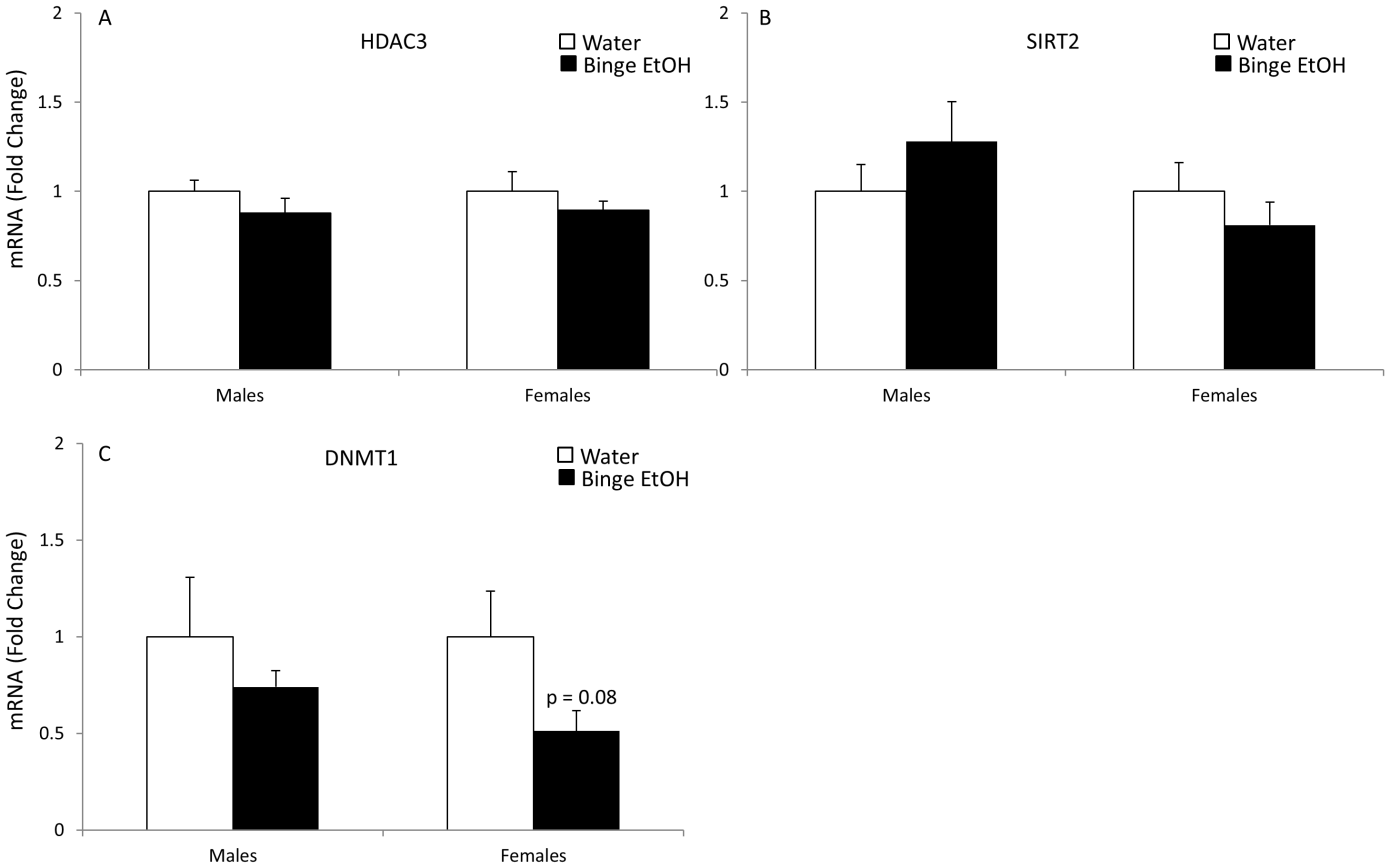
Effects of parental adolescent binge EtOH exposure on the expression of hypothalamic genes involved in chromatin modifications in the hypothalamus of PND 7 male and female offspring. qRT-PCR analysis of HDAC3 (A), SIRT2 (B), DNMT1 (C) mRNA expression in the hypothalamus of PND 7 male and female pups from water- or binge EtOH - treated parents (white and black bars, respectively). Data are expressed as mean ± SEM of mRNA fold change in relation to control pups from water- treated parents. (*) indicates a statistically significant difference from control (p<0.05).

Quantitative real-time RT-PCR was used to validate a subset of genes that were significantly altered according to the microarray analysis in PND 7 offspring of binge EtOH-exposed parents. Importantly, the qRT-PCR method was chosen to validate these genes because the sensitivity of detection for qRT-PCR is superior to a chip-based microarray platform. Out of those 17 selected genes, 70% showed the same results using both methods. More specifically, 65% showed similar results in males and 76% in females. The genes chosen for further analysis were selected based on their well described roles in neurodevelopment, neuroplasticity, and epigenetic processes including: [Fig. 2: fibroblast growth factor 13 (FGF13), bone morphogenic protein 1 (BMP1), reelin (RELN), serpin peptidase inhibitor 1 (SERPINI1)]; [Fig. 3: p21-activated kinase 3 (PAK3), insulin-like growth factor receptor 2 (IFG2R), vesicle associated membrane protein 3 (VAMP3), fibroblast growth factor receptor 3 (FGFR3)]; [Fig. 4: gonadotropin-releasing hormone (GnRH), apolipoprotein E (ApoE)]; [Fig. 5: early growth response 2 (Egr2), DICER1, WW domain binding protein 4 (WBP4), and small ubiquitin-like modifier protein 2 (SUMO2)]; [Fig. 6: histone deacetylase 3 (HDAC3), Sirtuin 2 (SIRT2), DNA methyltransferase 1 (DNMT1].

### Selected Genes in the Neurodevelopment Functional Cluster

Fibroblast growth factor 13 (FGF13) is an important regulator of neuronal polarization and migration [32]. Our results showed that parental adolescent binge EtOH exposure significantly decreased *Fgf13* hypothalamic mRNA expression in both male and female PND7 offspring (Fig. 2A). Another gene involved in neurodevelopment is BMP1, which interacts with other proteins to control neuronal stem cell differentiation [33]. Our qRT-PCR analysis showed a significant upregulation of *Bmp1* mRNA in male, but not female, offspring (Fig. 2B). Reelin (*Reln*), a large extracellular matrix protein that when secreted serves as a signalling molecule in cell-cell interactions, has been shown to play a role in the lamination of the hippocampal dentate gyrus [34], [35]. Our results revealed that there was a significant increase in *Reln* mRNA expression in female, but not male, offspring of parents exposed to adolescent binge alcohol (Fig. 2C). Finally, we analyzed the expression of *Serpini1*, which is a member of serpin superfamily of serine proteinase inhibitors that is secreted by axons and plays a role in regulation of axonal growth and synaptic plasticity [36], [37], [38]. Our qRT-PCR analysis revealed that *Serpini1* mRNA was significantly upregulated in F1 generation males due to parental adolescent binge EtOH exposure (Fig. 2D) and there was a similar trend in females.

Interestingly, a number of genes important for regulating synaptic plasticity were also identified by the microarray analysis as significantly altered due to paternal adolescent binge EtOH exposure including PAK3, IGF2R, VAMP 3, and FGFR3. According to our results, *Pak3* mRNA was significantly decreased by parental adolescent alcohol exposure in both F1 generation PND 7 males and females (Fig. 3A). Together with *Pak3*, IGF2R is also involved in spine maturation. There was a statistically significant increase in the expression of *Igf2R* mRNA in F1 generation PND 7 males (Fig. 3B) and a strong trend towards an increase in the expression of *Igf2R* mRNA in females (Fig. 4B, p = 0.099) due to parental adolescent binge EtOH exposure. Another protein important for synaptic plasticity is VAMP3, a SNARE complex interacting protein responsible for vesicle docking at the synapse [39]. Our qRT-PCR analysis showed that *Vamp3* mRNA was significantly increased in male F1 generation offspring pups after parental adolescent binge EtOH exposure (Fig. 3C). By contrast, there was an opposite effect in females which showed a decreased trend for *Vamp3* mRNA expression, although this result was not statistically significant (Fig. 3C). Finally, we analyzed the hypothalamic expression of *FgfR3* based on our initial microarray results. Previous work has shown that activation of *FgfR3* influences development of the occipitotemporal cortex during early postnatal life by overproduction of intermediate neuronal progenitors and therefore prolonged neurogenesis [40]. However, our qRT-PCR analysis did not show any significant changes in *FgfR3* mRNA levels in male or female offspring as a result of parental binge EtOH exposure (Fig. 3D).

### Selected Genes in the Metabolism Functional Cluster

One of the primary functions of the hypothalamus is the coordination of physiological homeostasis through regulation of feeding behaviors, reproduction, thermoregulation, and water balance. Two genes identified by microarray and cluster analyses were GnRH and ApoE. GnRH is the most upstream regulator of the hypothalmo-pituitary gonadal axis and is absolutely required for reproductive function. Moreover, the synthesis and release of GnRH is significantly elevated during pubertal maturation [41], [42], [43], [44], [45]. Our qRT-PCR results showed that *GnRH* was significantly increased, but only in F1 generation males due to parental adolescent binge alcohol exposure (Fig. 4A), and there was no effect in females. Perhaps the most striking results were observed with *ApoE*, a component of plasma lipoprotein that is involved in cholesterol metabolism and lipid transport [46]. *ApoE* is synthesized in the hypothalamus and plays a major role in regulation of feeding behavior [47]. Our qRT-PCR analysis showed that parental adolescent binge EtOH exposure almost completely abolished *ApoE* mRNA expression in both the male and female F1 generation offspring (Fig. 4B).

### Selected Genes in Transcriptional and Translational Regulation Cluster

Genes tested from this functional cluster included EGR2, DICER1, WBP4, and SUMO2. The early growth responsive 2 (*Egr2*) gene is a transcription factor that targets a number of genes involved in myelin formation and maintenance. Our results showed that *EgR2* mRNA expression was significantly decreased in female F1 offspring as a result of parental adolescent binge ETOH exposure (Fig. 5A). A similar sex difference was observed with *Dicer1*, which is important for posttranscriptional processing of small non-coding RNAs. Parental adolescent binge EtOH exposure significantly increased *Dicer1* in F1 male (Fig. 5B), but not female, offspring. Next, we measured *Wbp4*, a gene involved in alternative mRNA splicing [48]. Parental adolescent binge EtOH exposure significantly decreased *Wbp4* mRNA in the hypothalamus of F1 generation females (Fig. 5C), but males were unaffected. Finally, the posttranslational modifier, *Sumo2*, was increased by more than 2-fold in male offspring, but decreased by 30% in female offspring of parents exposed to adolescent binge EtOH (Fig. 5D).

### Genes in the Chromatin Modification Cluster

The last set of genes that we chose to further analyze using qRT-PCR were those involved in chromatin modifications. These genes are critical for regulating global gene expression patterns through alterations of histone proteins and nucleotides within gene promoter regions. For instance, HDAC3 is a protein that removes acetyl groups from histones, thereby making the DNA more accessible for transcriptional regulation [49]. The microarray analysis revealed that *Hdac3* was downregulated in both F1 generation males and females as a result of parental adolescent binge EtOH exposure, and our follow-up qRT-PCR analysis showed the same direction of change, in *Hdac3* mRNA expression, although it did not reach statistical significance (Fig. 6A). Another interesting histone deacetylase that came up on the microarray was the class III histone deacetylase SIRT2. Similar to HDAC3, *Sirt2* mRNA was downregulated in both F1 generation males and females as a result of parental adolescent binge EtOH exposure according to the microarray results. By contrast, however, our qRT-PCR analysis showed the same direction of change in mRNA expression of female offspring only (Fig. 6B). Lastly, DNMT1 is a maintenance methyltransferase and it is responsible for long-term gene methylation and therefore gene suppression. The microarray analysis showed that *Dnmt1* mRNA was upregulated in both F1 generation males and females however, this result could not be confirmed using qRT-PCR (Fig. 6C). Although there was no significant change in the expression of *Dnmt1* mRNA in the offspring of either sex, there was a 20% decrease in the expression observed in males and a strong trend towards a significant decrease in females (Fig. 6C, p = 0.086).

## Discussion

Adolescent binge alcohol abuse is an increasing societal burden. While it has been well documented that maternal alcohol abuse during pregnancy results in detrimental effects in the developing offspring, less is understood about heavy and repetitive alcohol abuse prior to, but outside of the gestational period, on neurodevelopment in the offspring. Moreover, the contribution of prior paternal (male) binge alcohol abuse on gene expression in the developing fetal brain is unclear. On the other hand, maternal exposure to environmental factors such as stress, drugs of abuse, and endocrine disruptors have been shown to affect gene expression patterns and behavioral responses in the offspring [1], [4], [5], [6], [50], [51], [52] and alcohol is both a drug of abuse and a physiological stressor, since it potently activates the hypothalamo-pituitary adrenal (HPA) axis [14], [15], [18], [53]. For example, early life stress in female mice has been associated with the development of a depressive-like phenotype in adulthood; a phenotype which persists in the future offspring, despite normal rearing conditions [4]. Moreover, child abuse in humans leads to an altered adult neuroendocrine response of the HPA axis that is perpetuated in the babies of female child abuse victims [54]. There is also some evidence that paternal exposure to other drugs of abuse can also adversely affect future offspring brain development. For instance, He and colleagues demonstrated significant impaired memory formation in the offspring of mice whose male, but not female, parent was exposed daily to cocaine beginning during early adolescence and continued daily until mating. Interestingly, the effect on memory impairment was permanent throughout the life of the offspring and was significantly greater in the female offspring [55]. The data presented in this report provide evidence that parental (both maternal and paternal) adolescent binge EtOH exposure, outside of fertilization or gestation periods, can lead to aberrant gene expression in the hypothalamus of the offspring. Specifically, these data show that parental adolescent binge EtOH exposure changed the expression of genes that are critical for neurodevelopment and synaptic plasticity (FGF13, BMP1, SERPINI1, RELN, PAK3, IGF2R, VAMP3, FGFR3), metabolic functions (GNRH, APOE), transcriptional regulation and posttranslational processing (EGR2, DICER1, WBP4, SUMO2) and chromatin modifications (HDAC3, SIRT2, DNMT1). Alteration in the expression of these genes during the fetal and early postnatal periods may lead to abnormal hypothalamic development and function, as well as increase the potential risk for mood disorders, stress-induced obesity, and addiction in adulthood.

### Paternal Adolescent Binge EtOH Exposure Significantly Altered Genes Critical for Neurodevelopment in the F1 Generation Offspring

Proper neurogenesis and neural differentiation during embryonic and early postnatal life is critical for healthy brain development and adult cognitive functions. We observed in these studies that parental adolescent binge EtOH exposure resulted in alterations of the expression of several genes critical for neurodevelopment (BMP1, FGF13, SERPINI1 and RELN) and synaptic functioning in the hypothalamus (IGF2R, FGFR3, PAK3, VAMP3). Growth factors, in particular, are critical for the formation and maintenance of the brain during embryogenesis. In our study, parental adolescent binge EtOH exposure significantly decreased the expression of *Fgf13* mRNA in the F1 generation offspring leading us to speculate that parental binge EtOH consumption may lead to abnormal hypothalamic development by decreasing critical growth factors important for embryonic brain development. Importantly, FGF13 is highly expressed in the brain and involved in tyrosine phosphorylation of mitogen-activated protein kinase (MAPK), c-RAF activation, phosphorylation of phospholipase C-gamma (PLCγ), increased number of glutamic acid decarboxylase positive neurons, increased GABA uptake, and increased choline acetyltransferase enzyme activity [56], all of which are important for successful neurodevelopment during embryogenesis. In addition, it has recently been shown that FGF13 is crucial for neuronal polarization and migration and that it acts as microtubule stabilizing protein during early development [32].

Similarly, another growth factor that was significantly altered in the F1 generation offspring in our study was BMP1. Our results showed that *Bmp1* mRNA was upregulated in PND 7 males as a result of parental adolescent binge EtOH exposure. The significance of increased *Bmp1* mRNA is currently unclear, however, one possibility might be that *Bmp1* upregulation is a compensatory effect to minimize neuronal damage. For instance, one of the primary functions for BMP1 is to regulate neural stem cell proliferation, differentiation and maturation of the developing brain, as well as stem cell maintenance in the subventricular zone of the adult brain [33], [57], [58].

Growth factor receptors are central components of growth factor signalling pathways and altered receptor complement can be equally deleterious as changes in ligand for growth factor activity. Our data revealed significant parental alcohol-induced increases in *Igf2R* mRNA expression in both male and female offspring, as well as decreased expression of *FgfR3*. Interestingly, IGF2R is important for regulating axonal spine maturation and synapse formation [59]. One possibility is that high IGF2R levels may be an indication of decreased IGF2R ligand availability, as many receptor:ligand concentrations are inversely correlated. In that case, it can be inferred that synapse formation in the offspring might be impaired as a consequence of paternal adolescent binge EtOH exposure. Similarly, FGFR3 has also been implicated in coordinating growth and development of the occipitotemporal cortex [40] and our data showed a significant decrease in *Fgfr3* mRNA expression in female pups after parental adolescent binge ETOH exposure. These data further support our hypothesis that binge drinking during adolescence could disrupt normal hypothalamic neurodevelopment in future offspring. Moreover, our data also demonstrate the interesting observation that parental adolescent exposure to EtOH induced sexually dimorphic changes in offspring gene expression profiles.

Our data revealed EtOH-induced changes in several other genes important for neurodevelopment in the offspring, which are not categorized specifically as growth factors. These include *Serpini1* (also known as neuroserpin) and *Reelin*. SERPINI1 is expressed in the brain where it inhibits tissue plasminogen and regulates axonal growth [60], [61]. Our data revealed that *Serpini1* mRNA was increased in the hypothalami of both male and female F1 generation PND 7 pups whose parents were prior exposed to adolescent binge EtOH. Interestingly, upregulation of *Serpini1* has also been linked with the pathogenicity of AD [37]. Similarly, our data showed that *Reln* mRNA expression was increased in female pups after parental adolescent binge ETOH exposure. REELIN is an extracellular matrix protein that controls neuronal migration and lamination of the hippocampus [34], [62], [63]. REELIN exerts its actions through *α*3β1 integrin to inhibit neuronal migration [63], suggesting that increased expression of *Reln* mRNA may lead to increased inhibition of neuronal migration in the hypothalamus and therefore, disrupted hypothalamic neuronal connectivity.

Successful neurodevelopment also requires the formation of appropriate synaptic connections and maintenance of coordinated synaptic activity. We found that parental adolescent binge EtOH exposure results in altered expression of a variety of genes that regulate synaptic plasticity, including PAK3. The p21-activated kinase 3 (PAK3) is known to act through the CDC42/Rho pathway and is implicated in regulating various neuronal functions including synaptic plasticity and spine morphogenesis [64], cytoskeletal dynamics, as well as cell proliferation and differentiation [65]. Mutations in the expression of the PAK3 gene and abnormalities in spine maturation have been correlated with mental retardation [64], [66], [67], [68]. Our data showed that there was a significant decrease in *Pak3* mRNA levels after parental adolescent binge EtOH exposure in both male and female F1 generation PND 7 pups, suggesting that the fine-tuning processes associated with brain maturation could be adversely affected. The DAVID functional cluster analysis also showed that genes involved in SNARE interactions with vesicular and secreted proteins, such as VAMP3, were also altered by parental binge EtOH exposure. VAMP3 is a fusion protein associated with SNARE complexes, highly expressed in astrocytes, and facilitates vesicle docking at the membrane [39]. Activation of VAMP3 has been shown to influence glutamate and D-serine release [69] therefore, increased Vamp3 mRNA expression as observed in our study may indicate a potential for abnormal synaptic functioning.

### Paternal Adolescent Binge EtOH Exposure Altered Genes that Regulate Metabolism in the F1 Generation Offspring

The hypothalamus is the primary brain region mediating homeostasis through its central regulation of circadian rhythms, reproduction, feeding behaviors, thermoregulation, osmotic balance and stress responses. One of the most striking observations in our study was the complete abolition of *ApoE* mRNA expression in both male and female F1 generation offspring as a result of parental adolescent binge EtOH exposure. *ApoE* is involved in lipid metabolism and cholesterol homeostasis [46]. Recent data has shown that *ApoE* is regulated in the arcuate nucleus of the hypothalamus by the hormone leptin, which regulates feeding behavior. [70]. Although we did not observe any birth weight differences in the offspring from control and EtOH-exposed parents, a significant decrease in *ApoE* expression in the offspring could potentially predispose them for an increased risk of developing obesity later in life. Another significant finding was the observed increase in *GnRH* mRNA expression in male offspring as a result of parental adolescent binge EtOH exposure. *GnRH* has long been known for stimulation of gonadotropin release from the pituitary gland in both males and females and therefore, is absolutely required for pubertal onset and fertility [43], [44], [45], [71], [72], [73], [74], [75], [76], [77], [78], [79], [80]. Recently, a role for *GnRH* in metabolic functions has been demonstrated by Harris and colleagues, who showed that *GnRH* increased the expression of the glucose transporter, GLU-1, in pituitary gonadotrophs and stimulated glucose uptake in these cells [81]. The observed significant increase in *GnRH* expression in the male offspring could lead to abnormal pituitary glucose regulation, thereby raising the potential for a potential host of neuroendocrine dysfunctions.

### Paternal Adolescent Binge EtOH Exposure Altered Genes Important for Modifying Chromatin, Transcriptional Regulation, and Posttranslational Processing in the F1 Generation Offspring

Altered gene expression can result from modifications in the transcriptional machinery, such as through chromatin modifications and transcription factors, or posttranslational processing. In this study we did not analyze specific epigenetic modifications of chromatin for any of the genes tested. However, an epigenetic transfer from the parental generation is the most parsimonious explanation for the effects observed in the offspring, which were not exposed to any differential conditions in utero. In this study, we demonstrate that the F1 generation offspring of parents exposed to binge EtOH during adolescence had significantly different gene expression profiles according to the microarray analysis for genes that are capable of modifying chromatin structure (HDAC3, SIRT2, DNMT1) genes that regulate transcription and RNA processing (EGR2, WBP4, DICER1), and genes that regulate posttranslational modifications of proteins (SUMO2). Importantly, however, only *Egr2* and *Wbp4* were statistically significant when validated using qRT-PCR. This could be due to amplification of individual variance that was observed using the highly sensitive qRT-PCR method and our relatively low sample size (N = 6). Nevertheless, the results obtained using qRT-PCR had the same trends and direction of change as the results using microarray analysis, suggesting that these results could be biologically relevant. HDAC3 is a histone deacetylase that removes acetyl groups from the histone making the DNA more tightly bound to the histone proteins and therefore, less accessible for transcriptional regulation. In general, it is well accepted that HDAC3 suppresses global gene transcription in a wide variety of systems. However, it has also been shown to have very specific roles in the regulation of normal brain function. For instance, HDAC3 acts in concert with nuclear receptor corepressors (such as NCor and SMRT), to regulate thyroid hormone receptor [82], neurogenesis [83], as well as for the suppression of long term memory formation [84]. Our data showed that *Hdac3* mRNA was downregulated in the hypothalami of both male and female PND 7 pups whose parents were exposed to adolescent binge EtOH. A decrease in *Hdac3* expression could have an overall positive impact on gene transcription and might explain the increased expression levels we observed in several other genes. In addition to alterations in *Hdac3* expression, we also observed gene expression changes in another type of histone deacetylase, SIRT2, which is a NAD-dependent class III histone deacetylase. Similar to other deacetylases, SIRT2 is primarily involved in transcriptional silencing [85], [86], [87]. Chronic cocaine exposure increased the catalytic activity of SIRT2 in the nucleus accumbens and a pharmacological block of SIRT2 activity dramatically reduced cocaine-seeking behavior, suggesting that SIRT is an important component of the reward pathways for drugs of abuse [86].

Our studies showed a significant decrease in *Egr2* mRNA expression in female F1 offspring of parents exposed to binge EtOH. The potential consequences of this for female offspring is unclear however, mutations in this gene cause severe sensory deficits in human patients and is associated with Charcot Marie Tooth Syndrome [88]. EGR2 is a transcription factor that regulates transcription of a variety of genes, particularly those involved with axonal myelination. Interestingly, increased *Egr2* mRNA expression in Fos-positive neurons of the prefrontal cortex has also been linked with cue-induced relapse to drug addiction [89]. One of the more interesting genes that we found altered in our study was *Dicer1*, which regulates small noncoding RNA processing including microRNAs [90]. microRNAs have recently come to the forefront as a critical molecular mechanism underlying virtually all cellular process. Notably for our study, DICER1 has been shown to mediate stress-induced anxiety through its regulation of microRNA-34 in the amygdala [91]. These data raise the interesting possibility that changes in *Dicer1* mRNA expression in male offspring after parental binge EtOH exposure may have far-reaching effects on posttranscriptional gene regulation. Two other interesting genes that we investigated regulate gene expression through transcriptional or posttranslational modifications (WBP4 and SUMO2). *Wbp4* encodes a spliceosomal domain that is involved in binding various proline rich ligands and influences pre-mRNA splicing [48], [92], [93], [94]. SUMO2, on the other hand, is a protein that conjugates lysine residues of target proteins and modulates protein activity, stability and cellular localization. Interestingly, the main targets for SUMO proteins are transcription factors that regulate gene transcription and expression [95], [96], [97]. Changes in these genes after parental binge EtOH consumption point to yet another mode of possible mechanisms responsible for altered gene transcription in the offspring.

### Conclusions

Taken together, our data herein describe highly novel results showing significant changes in hypothalamic gene expression in offspring born to parents exposed to adolescent binge EtOH. Children of alcoholic parents are more prone to develop psychological disorders, such as anxiety, major depression, conduct abnormalities, and attention deficit disorder [98], [99], [100], [101], [102], [103], [104]. Moreover, adoption and twin studies have demonstrated that the propensity to develop these psychological disorders has a strong genetic component [100], [105] however, a clear genetic mark has yet to be identified. Those findings raise the possibility that EtOH exposure could induce epigenetic modifications in the parents resulting in an epigenetic “signature” that is transmitted to the offspring. However, we acknowledge that caution must be exercised when drawing conclusions based on gene expression studies. For instance, changes in gene expression can be a predisposing factor for the development of neurological and behavioral disorders, although both the positive and negative effects of environment cannot be discounted. In our study, the mothers were not intoxicated at any point during mating or pregnancy leading to the assumption that their behavior during pregnancy, and also postnatal, was similar to controls. Despite this assumption, physiology ultimately dictates behavior therefore, identifying the molecular basis of the physiological changes in the parent is critical to understanding the transgenerational effects of adolescent binge alcohol exposure. In addition, the maternal uternine environment might have also been a factor. The experimental approach for these studies exposed both the male and female parents to our binge EtOH paradigm making it impossible to determine whether one parent conferred more epigenetic information than the other and to date, we understand very little about the mechanisms for how epigenetic marks are conferred to offspring. In addition, we used a “two-hit” paradigm in which animals were exposed to repeated binge EtOH during both the early (37–44 days of age) and late (67–72 days of age) pubertal period, which was designed to mimic early and late human adolescent exposure (13–16 & 18–21 yrs). Therefore, the precise window of vulnerability during adolescence remains unclear. Our previous studies showed that perturbations in the HPA axis were observed only with an early adolescent binge and not with a single late adolescent “binge”, indicating the early adolescent binge was critical [14]. Therefore, we would predict that a single binge episode during the early adolescent period would be sufficient to elicit the observed altered gene expression in the offspring, however, the importance of late adolescent binge EtOH exposure cannot be ruled out at this time. Overall, these data are a first step towards fully understanding the long-term and transgenerational consequences of adolescent binge alcohol abuse.
